# An engineered membrane-bound guanylyl cyclase with light-switchable activity

**DOI:** 10.1186/s12915-021-00978-6

**Published:** 2021-03-29

**Authors:** Yuehui Tian, Georg Nagel, Shiqiang Gao

**Affiliations:** 1 Department of Neurophysiology, Institute of Physiology, Biocenter, University of Wuerzburg, 97070 Wuerzburg, Germany; 2 Present address: Environmental Microbiomics Research Center, School of Environmental Science and Engineering, Southern Marine Science and Engineering Guangdong Laboratory (Zhuhai), Sun Yat-sen University, Guangzhou, 510006 China

**Keywords:** *Chlamydomonas reinhardtii*, Guanylyl cyclase, Optogenetics, Rhodopsin, Cyclic GMP

## Abstract

**Background:**

Microbial rhodopsins vary in their chemical properties, from light sensitive ion transport to different enzymatic activities. Recently, a novel family of two-component Cyclase (rhod)opsins (2c-Cyclop) from the green algae *Chlamydomonas reinhardtii* and *Volvox carteri* was characterized, revealing a light-inhibited guanylyl cyclase (GC) activity. More genes similar to 2c-Cyclop exist in algal genomes, but their molecular and physiological functions remained uncharacterized.

**Results:**

Chlamyopsin-5 (Cop5) from *C. reinhardtii* is related to *Cr*2c-Cyclop1 (Cop6) and can be expressed in *Xenopus laevis* oocytes, but shows no GC activity. Here, we exchanged parts of Cop5 with the corresponding ones of *Cr*2c-Cyclop1. When exchanging the opsin part of *Cr*2c-Cyclop1 with that of Cop5, we obtained a bi-stable guanylyl cyclase (switch-Cyclop1) whose activity can be switched by short light flashes. The GC activity of switch-Cyclop1 is increased for hours by a short 380 nm illumination and switched off (20-fold decreased) by blue or green light. switch-Cyclop1 is very light-sensitive and can half-maximally be activated by ~ 150 photons/nm^2^ of 380 nm (~ 73 J/m^2^) or inhibited by ~ 40 photons/nm^2^ of 473 nm (~ 18 J/m^2^).

**Conclusions:**

This engineered guanylyl cyclase is the first light-switchable enzyme for cGMP level regulation. Light-regulated cGMP production with high light-sensitivity is a promising technique for the non-invasive investigation of the effects of cGMP signaling in many different tissues.

**Supplementary Information:**

The online version contains supplementary material available at 10.1186/s12915-021-00978-6.

## Background

Microbial rhodopsins are important photoreceptors for microorganisms such as archaea, bacteria, giant viruses [1] and lower eukaryotes including algae, fungi, and choanoflagellates [2, 3]. The different rhodopsins function as light sensors with very different output function.

As an active light-responsive microorganism, the green alga *Chlamydomonas reinhardtii* was predicted to contain about 10 opsin genes in the genome [4, 5], some of them with hypothetical cyclase activity. Only after covalent binding of the opsin to the light-absorbing chromophore all-trans retinal (vitamin A) the protein becomes light-sensitive and functional; it is then called rhodopsin. However, until now, only three rhodopsin molecules are functionally characterized [6–8] and two of them with confirmed physiological function [9]. The first hypothesized “opsins,” Chlamyopsin-1 and 2 (Cop1, Cop2) [10], show no homology to the other authentic *Chlamydomonas* opsins and are not transmembrane proteins [8, 11] and therefore should not be regarded as “opsins.” Their *Volvox carteri* homolog Vop1 was exclusively found at the oldest basal bodies of the embryo and on the corresponding d-roots and was renamed “basal body protein-1” (Babo1 [11]). Cop3 and Cop4 (originally named CSRA and CSRB for “Chlamydomonas Sensory Rhodopsins A and B”) were the first two authentic *Chlamydomonas* opsins, shown to function physiologically as phototaxis receptors of *C. reinhardtii* [9]. The molecules Cop3 and Cop4 were characterized after heterologous expression as direct light-gated cation channels and named Channelrhodopsin-1 and 2 [6, 7]. We recently characterized the activity of a very large opsin from *C. reinhardtii: Cr*2c-Cyclop1 (Cop6). *Cr*2c-Cyclop1 was determined as ATP-dependent and light-inhibited guanylyl cyclase with an action spectrum peaking at ~ 540 nm [8]. Also, the *V. carteri* homolog is large and shows similar function [8].

In contrast to the classical 7-TM (seven transmembrane helices) topology of rhodopsins, the opsin domain of *Cr*2c-Cyclop1 contains eight transmembrane helices, with cytosolic N- and C-terminus, as all other so far identified enzyme rhodopsins, like the light-activated guanylyl cyclase from *Blastocladiella emersonii*—*Be*Cyclop [12–15]—and the light-sensitive phosphodiesterase RhoPDE [16]. Interestingly, all three classes of microbial enzyme rhodopsins, discovered so far, are related to regulation of cGMP concentration by light. As these light-regulated enzymes were shown to be functionally expressed in mammalian cells, they are promising tools in cGMP research and are even considered as possible candidates for gene therapy.

Another opsin with homology to *Cr*2c-Cyclop1, Cop5 from *C. reinhardtii*, has a similar domain architecture as *Cr*2c-Cyclop1. But expression of Cop5 did not yield guanylyl cyclase (GC) activity [8]. This might be because of its GC domain which lacks several key residues, playing roles in metal ion and GTP binding as well as in transition state-stabilization, required for cGMP production. If Cop5 plays a functional role in the alga, this function might result from a heterodimer formation with a yet unidentified cyclase domain in vivo. Although Cop5 could be expressed in the plasma membrane of oocytes from *X. laevis* [8], purification of the full-length protein was not achieved until now. However, the Cop5 rhodopsin part (HKR1) was purified after heterologous expression in the methylotrophic yeast *Pichia pastoris* and studied with spectroscopic methods. It was determined as an UV-A receptor with an absorption spectrum peaking at 380 nm. Its two isoforms Rh-UV and Rh-Bl are finely tuned by UV-A and blue light and thermally stable in the dark. The excited state lifetimes of Rh-UV and Rh-Bl were shown to be greater than 1 h [17–20].

In this study, we aimed to determine if (and which) parts of Cop5 are able to transmit an absorbed light signal, by fusing them to the corresponding functional GC domain of *Cr*2c-Cyclop1. Therefore, we generated chimeras between Cop5 and *Cr*2c-Cyclop1. We obtained a bi-stable two-component cyclase opsin (switch-Cyclop), which can be switched on for hours by a short 380 nm light flash and then switched off with blue or green but not with red light. The ratio of GC activity in the activated state to activity in the inhibited state is up to 20. This designed two-component cyclase opsin is very sensitive to UV-A and blue light. After a short UV-A stimulation, the GC activity is stable for at least 6 h in the dark, which can then be inhibited by a short blue or green light pulse. The engineered switch-Cyclop should be useful to regulate cGMP concentration in different cells or live animals by switching between UV-A and blue/green light pulses.

## Results

### The opsin domain of Cop5 enables light color-signaling

Our previous study showed that Cop5 can be expressed in oocytes of *X. laevis* but that no guanylyl cyclase activity can be detected whereas Cop6 (*Cr*2c-Cyclop1) was demonstrated to be a light-inhibited guanylyl cyclase [8]. Sequence alignment shows that the GC domain of Cop5 is missing several key residues required for metal ion binding, base recognition, ribose-orienting, and transition state-stabilization in the cGMP production process (Additional file 1: Figure S1). Therefore, three chimeras of Cop5 and *Cr*2c-Cyclop1 were designed for fusion of corresponding fragments at regions of high homology and outside predicted domains. All three chimeras retained the complete opsin part of Cop5.

**Fig. 1:**
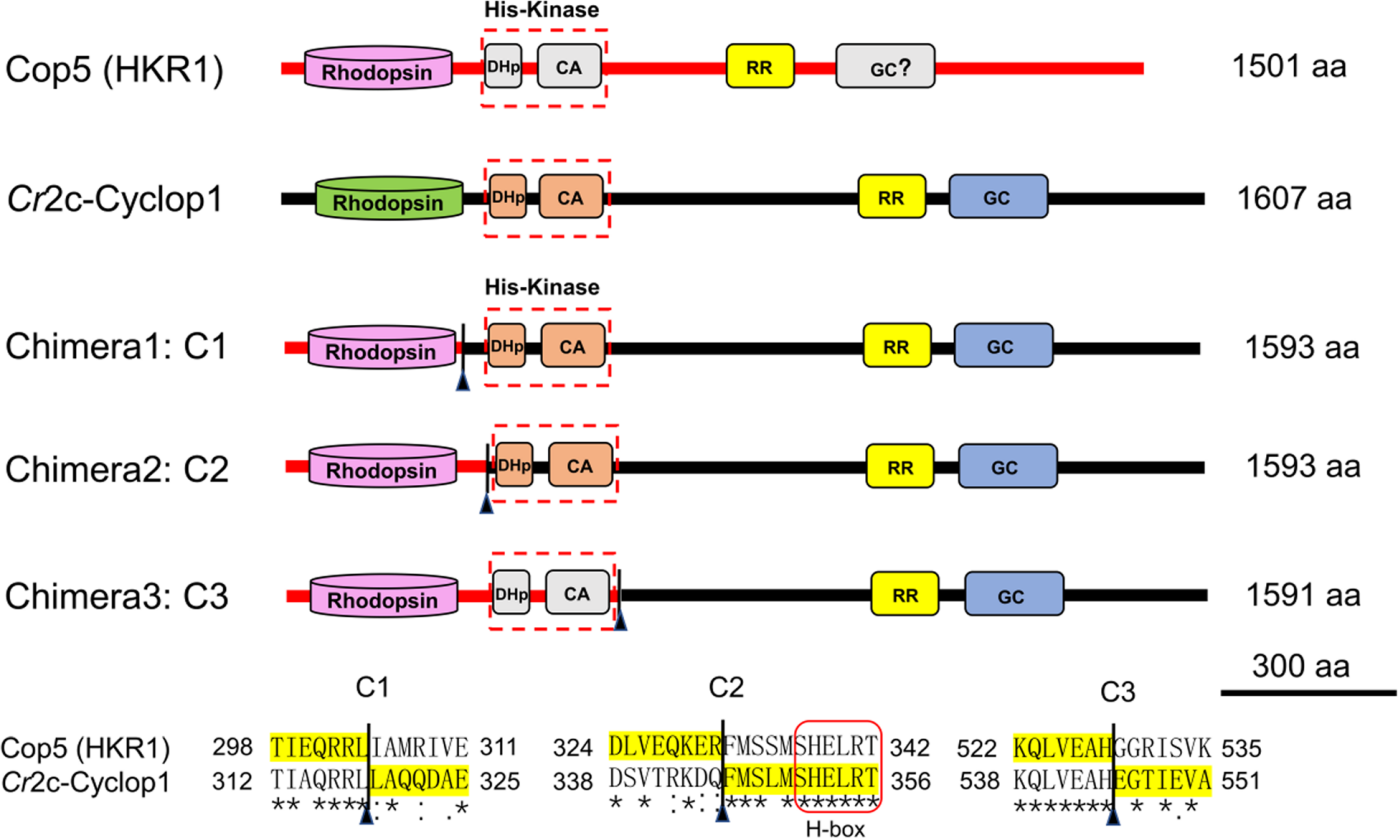
Schematic model of Cop5, *Cr*2c-Cyclop1 and designed chimeras. Colored modules indicate diverse protein domains of Cop5 (Cre02.g074150, 1501 aa) and *Cr*2c-Cyclop1 (Cre11.g467678, 1607 aa). Rhodopsin, rhodopsin domain; DHp, dimerization and histidine phosphotransferase domain; CA, catalytic and ATP-binding domain; RR, response regulator domain; GC, guanylyl cyclase domain. His kinase domain is labeled in dashed red boxes. In three chimeras C1, C2, and C3, the fusion positions were indicated by black line with triangle, amino acid sequences of the fusion constructs were highlighted with yellow color. H-box domain of the His Kinase is marked with red frame

The chimeras were fused:

1. After the opsin domain of Cop5 (Chimera 1, C1),
2. Before the histidine kinase domain of *Cr*2c-Cyclop1 (Chimera 2, C2),
3. After the histidine kinase domain of Cop5 (Chimera 3, C3) (Fig. 1).

**Fig. 2:**
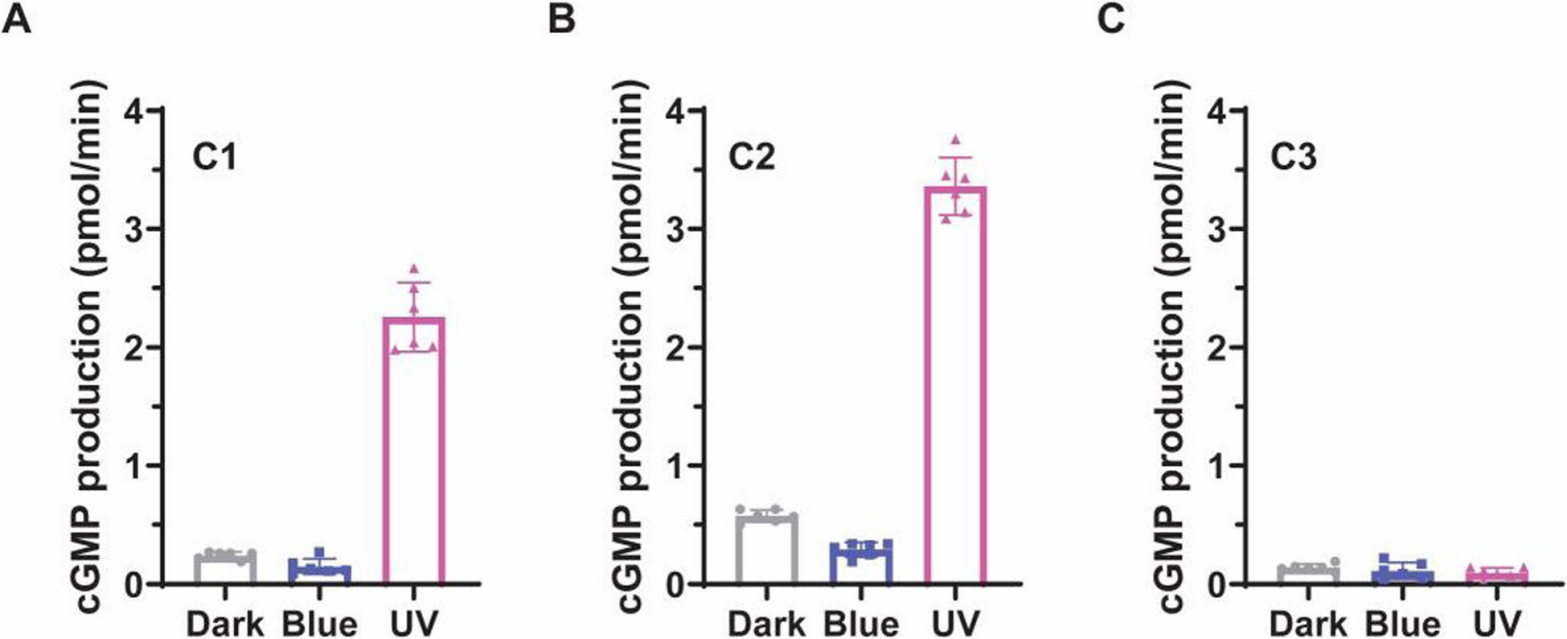
Light-regulated enzymatic activities of chimeras 1–3. Enzyme activity of C1 (**a**), C2 (**b**), and C3 (**c**) under constant dark, Blue (blue light, 473 nm, 50 μW/mm^2^) and UV (UV-A light, 380 nm, 2.4 μW/mm^2^). Thirty nanograms of cRNA were injected for each; measurements were done 3 dpi (days post injection). Final results were referring to the total activities of membrane proteins from one oocyte. *n* = 6, error bars = SD

The chimeras were expressed in *X. laevis* oocytes and guanylyl cyclase (GC) activities of membrane extracts were measured in an established in-vitro assay [8, 12, 16]. Chimeras C1 and C2 showed a low GC activity in the dark and in 473 nm light but higher GC activity with 380 nm illumination (Fig. 2a and b). The ratio of activity under these preliminary test conditions of UV-A and blue illumination was determined to be 15 and 12 for C1 and C2, respectively.

Chimera C3 showed only a low GC activity, compared to C1 and C2, with no difference between the dark, UV-A, and blue light conditions (Fig. 2c). This implies that the His kinase domain from Cop5 is not functional or not compatible with the response regulator domain of *Cr*2c-Cyclop1. For further investigation, we focus on C1 because of its light-sensitivity with the highest activation ratio.

### The engineered chimera C1 is bistably regulated by UV-A and blue/green light

**Fig. 3:**
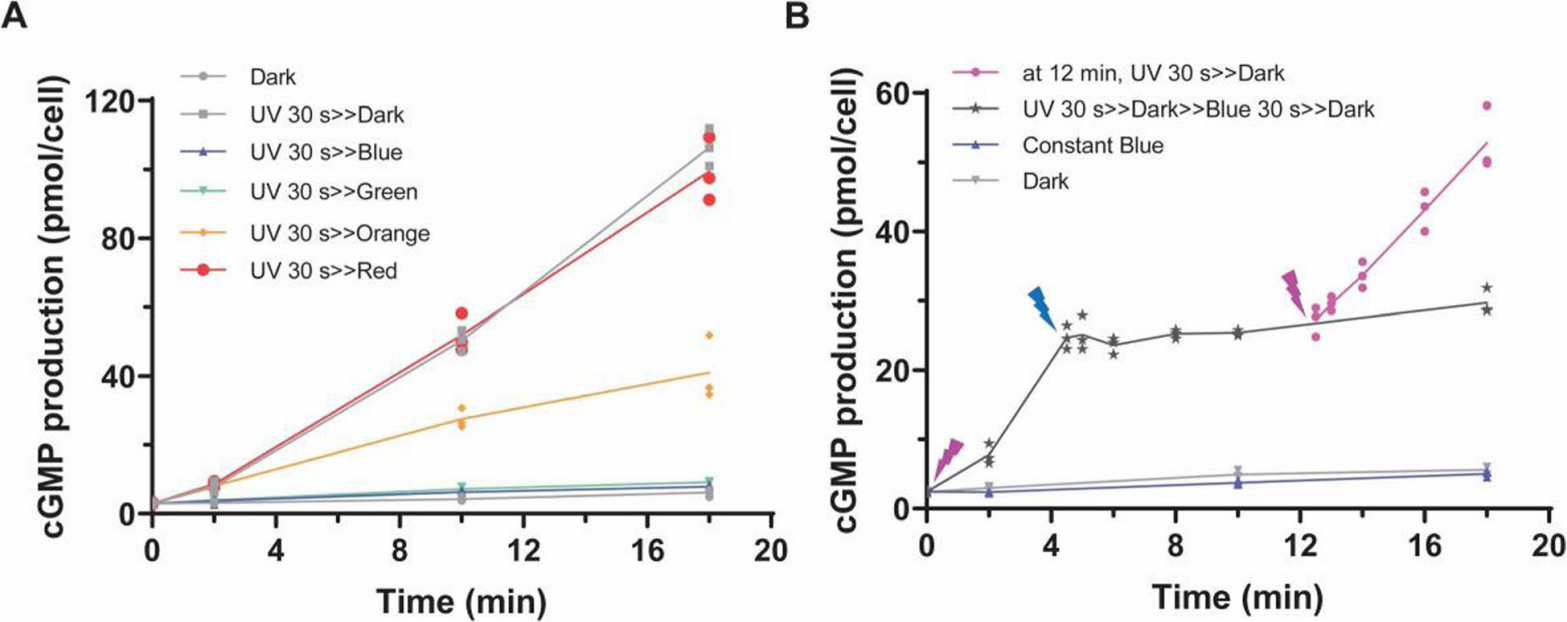
Dynamic regulation of switch-Cyclop1 (C1) activity by different light colours. **a** After 30 s UV-A (380 nm, 9.6 μW/mm^2^) illumination, cGMP productions were measured at different time points under dark or constant illumination with light of different wavelengths. Light intensities of blue (473 nm), green (532 nm), orange (593 nm), and red (635 nm) were all adjusted to 4.8 μW/mm^2^. *n* = 3, all data points were shown. **b** Dynamic control of switch-Cyclop1 activity by UV-A and blue light; 30 s UV-A light (380 nm, 9.6 μW/mm^2^) and 30 s blue (473 nm, 9.6 μW/mm^2^) illumination were used to activate and inhibit cGMP production. , 30 s UV-A at time 0, then dark, 30 s blue at 4 min, then dark, then 30 s UV-A at 12 min and then dark until 18 min; , 30 s UV-A at time 0, then dark, then 30 s blue at 4 min and then dark until 18 min; , constant blue until 18 min; , constant dark until 18 min. Measurements of cGMP production in the dark or under constant blue illumination were performed in parallel. For both **a** and **b**, 30 ng cRNA were injected; measurements were done 3 dpi. The results were referring to the total activities of membrane proteins extracted from one oocyte. *n* = 3; all data points were shown

After confirming the UV-activated GC activity of C1, we designed experiments to test whether C1 showed a bi-stable character, as might be predicted from the spectroscopic studies [17–20]. As shown in Fig. 3a, a short (30 s) 380-nm light pulse could activate the GC activity of C1 which continued in the dark. We then tested the inhibition effect with light of different wavelengths on UV-A-activated switch-Cyclop1. A 30-s (380 nm) UV-A illumination was applied in the beginning, and then the samples were either kept in the dark or in 473 nm blue light, 532 nm green light, 593 nm orange light, or 635 nm red light to measure GC activity. As shown before, the GC activity remained high in the dark. The UV-A-evoked GC activity can be efficiently turned down by 4.8 μW/mm^2^ 473 nm blue and 532 nm green light (Fig. 3a). A 593 nm light can only turn down the evoked GC activity partially while the 635 nm red light has no effect on UV-A-evoked GC activity, similar to the dark (Fig. 3a). After a short UV-A light pulse for 30 s, the evoked GC activity could then be switched off by 30 s 473 nm light similar with the GC activity in the dark, while the activation by UV-A can be repeated after the inhibition by blue light (Fig. 3b). Figure 3b shows that C1 is switched by UV-A and blue light between two activity states, in good accordance with results of the previous spectroscopic studies on the Cop5 rhodopsin domain [17–20]. Thus, we name the chimera C1 “switch-Cyclop1” (and C2 “switch-Cyclop2”), for bi-stable or “switchable cyclase opsin.”

The published study on the rhodopsin part of Cop5 (HKR1) revealed two light-switchable isoforms, Rh-UV (with absorption maximum at 380 nm) and Rh-Bl (with absorption maximum at 487 nm), which were suggested to be thermally stable in the darkness. Both isoforms can be efficiently interconverted by UV-A and blue light, and both states (Rh-UV and Rh-Bl) were shown to be absolutely stable for at least 50 min [17]. We determined the stability of enzyme activity in the dark after UV-A stimulation. We initially observed a gradual decrease of GC activity during incubation for several hours (Additional file 1: Figure S2A). However, it turned out that under our in vitro reaction conditions, addition of fresh ATP and GTP after 4 h completely restored the initial enzymatic activity (Additional file 1: Figure S2A). When fresh GTP and ATP were supplied every hour, the enzyme activity was constant in the dark for at least 6 h after UV-A- activation (Additional file 1: Figure S2B).

### Activity of switch-Cyclop1 is switched by weak light pulses

**Fig. 4:**
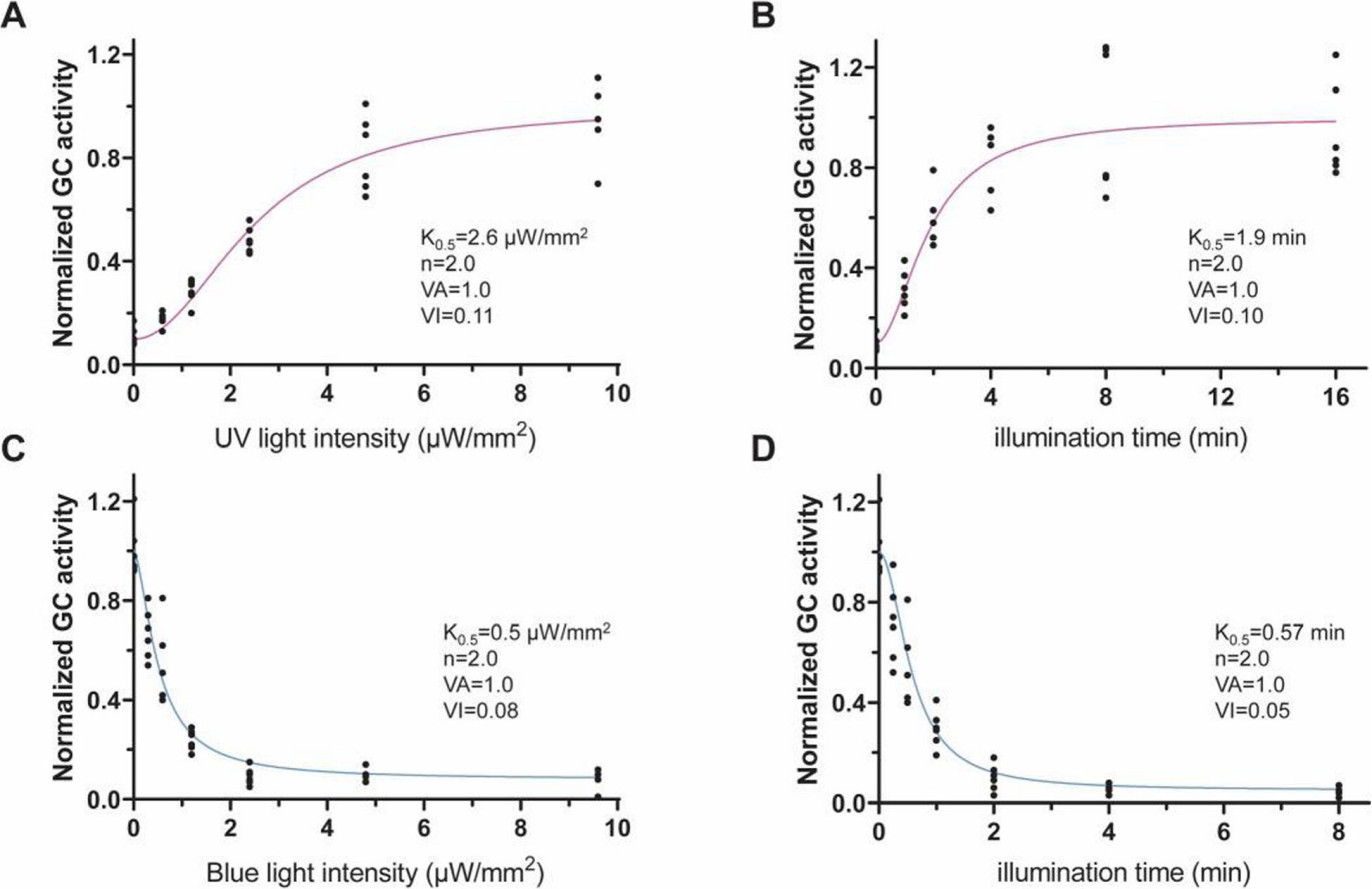
Illumination conditions required for switch-Cyclop1 regulation. **a** Enzyme activities after 30 s illumination with UV-A light of different intensities (0, 0.6, 1.2, 2.4, 4.8, 9.6 μW/mm^2^). A data point with 30 s 9.6 μW/mm^2^ UV-A illumination was set to “1” for normalization. Curve fitted with Hill1 equation. **b** Enzyme activities after different time (0, 1, 2, 4, 8, and 16 min) of illumination with 0.6 μW/mm^2^ UV-A. A data point with 8 min 0.6 μW/mm^2^ UV-A illumination was set to “1” for normalization. Curve fitted with Hill1 equation. **c** After 30 s 9.6 μW/mm^2^ UV-A (380 nm) light stimulation, 30 s blue light (473 nm) illuminations with different intensities (0.3, 0.6, 1.2, 2.4, 4.8, 9.6 μW/mm^2^) were used to inhibit the evoked GC activity. Curve fitted with Hill1 equation. **d** After 30 s 9.6 μW/mm^2^ UV-A (380 nm) light stimulation, 0.6 μW/mm^2^ blue light (473 nm) illumination of different time (0.25, 0.5, 1, 2, 4, and 8 min) were used to inhibit the evoked GC activity. Curve fitted with Hill1 equation. For **c** and **d**, one repeat of enzyme activity after 30 s UV-A light (380 nm, 9.6 μW/mm^2^) was set to “1” for normalization; 30 ng cRNA were injected for 3 days’ expression. For all, *n* = 6, all data points were shown

To determine the light conditions required for activation, we first applied UV-A light ranging from 0.6 to 9.6 μW/mm^2^ with a 30 s duration and then measured activity in dark for 2, 10, and 18 min. As shown in Fig. 4a, activity increased with the increasing light power. 9.6 μW/mm^2^ is near the saturation for a 30 s illumination. With 30 s illumination half-maximal activity was obtained with ~ 2.6 μW/mm^2^, corresponding to a K_0.5_ of 78 μJ/mm^2^ (= 78 J/m^2^).

At a given light intensity, the activation should also depend on the duration, as confirmed by UV-A illumination of low intensity (0.6 μW/mm^2^) with different time windows (Fig. 4b). The required time for 50% activation is about 1.9 min with 0.6 μW/mm^2^, which results in a K_0.5_ of 68 μJ/mm^2^ (= 68 J/m^2^). We therefore estimate the required energy density for half-maximal activation by 380 nm photons at 73 J/m^2^.

We further evaluated the blue light conditions required for inhibiting the UV-A-activated switch-Cyclop1. After activation with 30 s 9.6 μW/mm^2^ UV-A light, 30 s illumination with blue (473 nm) light ranging from 0.6 to 9.6 μW/mm^2^ was applied to inhibit the enzyme. As shown in Fig. 4c, 30 s 0.5 μW/mm^2^ blue light inhibits ~ 50% of active switch-Cyclop1, and 30 s 9.6 μW/mm^2^ can inhibit more than 90% of active switch-Cyclop1. When applying lower light power and longer times, we determined that 0.6 μW/mm^2^ inhibits ~ 50% of active switch-Cyclop with ~ 30 s illumination (Fig. 4d). These results yield a K_0.5_ for blue light-induced inhibition of 15 J/m^2^ and of 20 J/m^2^, respectively. The estimated energy density for half-maximal inhibition by 473 nm photons is therefore 18 J/m^2^.

Similar to *Cr*2c-Cyclop1, functionality of switch-Cyclop1 requires Mg^2+^ and ATP (Additional file 1: Figure S3). Mg^2+^ plays distinct roles in regulating nucleotidyl cyclase activity and ATP is required for auto-phosphorylation of the conserved histidine by the kinase domain. When replacing Mg^2+^ with Ca^2+^, the enzyme activity was highly decreased, similar to the blue-inhibited state (Additional file 1: Figure S3). Ca^2+^ therefore cannot replace Mg^2+^.

### Switch-Cyclop1 can sense different light ratios

Our results confirm the bi-stable regulation of the Cop5 Rhodopsin domain (HKR1) by UV-A and blue (or green) light, when fused to a domain with GC activity. However, in nature, there is always a mixture of photons of different wavelengths. We therefore tested how switch-Cyclop1 reacts to mixtures of 380 nm and 505 nm light.

**Fig. 5:**
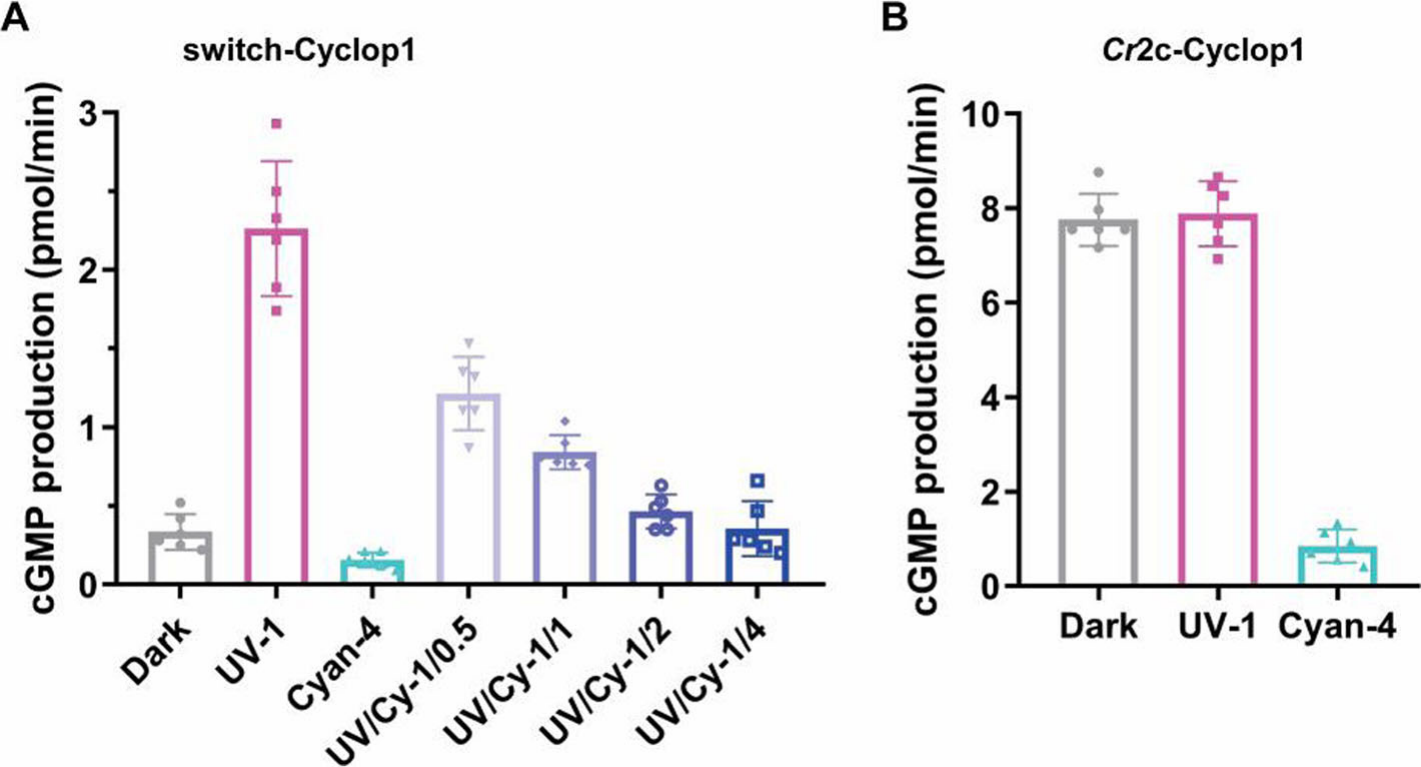
switch-Cyclop1 and *Cr*2c-Cyclop activities under different light conditions. Enzyme activities of switch-Cyclop1 (**a**) and *Cr*2c-Cyclop1 (**b**) under different light conditions. Reactions are done in the dark or under constant light illumination conditions. Illumination is performed with 380 nm UV-A and 505 nm cyan LED. Exact illumination conditions are UV-1 (UV-A 1 μW/mm^2^), Cyan-4 (cyan light 4 μW/mm^2^), UV/Cy-1/0.5 (UV-A 1 μW/mm^2^ + cyan 0.5 μW/mm^2^), UV/Cy-1/1 (UV-A 1 μW/mm^2^ + cyan 1 μW/mm^2^), UV/Cy-1/2 (UV-A 1 μW/mm^2^ + cyan 2 μW/mm^2^) and UV/Cy-1/4 (UV-A 1 μW/mm^2^ + cyan 4 μW/mm^2^); 40 ng cRNA were injected for each; measurements were done 3 dpi. The results were referring to the total activities of membrane proteins extracted from one oocyte. *n* = 6, error bars = SD

We used 1 μW/mm^2^ continuous UV-A light for activation and different intensities of 505 nm light were added in parallel experiments. The switch-Cyclop1 activity decreases with the increasing ratio of 505 nm light (Fig. 5a). switch-Cyclop1 is an artificial chimera but it utilizes the full rhodopsin domain of Cop5 for photo sensing. This suggests that the Cop5 rhodopsin (HKR1) can sense the cyan/UV-A ratio in the living alga, if the protein Cop5 is expressed and is functional (e.g. as a heterodimer).

Cop5 and *Cr*2c-Cyclop1 probably co-exist in *C. reinhardtii*, but their rhodopsin part shows quite different characteristics: Cop5 rhodopsin is bi-stable and *Cr*2c-Cyclop1 rhodopsin is light-inhibited. However, we found that UV-A light (at 1 μW/mm^2^) cannot activate or inhibit *Cr*2c-Cyclop1, as its activity in UV-A is not different to the activity in dark (Fig. 5b).

## Discussion

Up to seven opsin genes in the genome of *C. reinhardtii* were predicted already in 2004 [4, 5] and twelve were predicted in 2017 [4, 5], but only three of them have been proven functionally [6–8] at the molecular level and two of them were disproven as opsin proteins. The first functionally characterized authentic *Chlamydomonas* opsins were Cop3 and Cop4 (also named CSRA and CSRB [9]) and better known as channelrhodopsin-1 and channelrhodopsin-2 (ChR1 & ChR2) [6, 7]. The first “discovered” Chlamyopsins—Cop1 & Cop2 [8]—were recently found to be soluble proteins [8] and can be dismissed as opsins, reducing the number of possible opsins in *C. reinhardtii* to ten.

The light-inhibited two component-cyclase opsin, 2c-Cyclop1 [8], was the third functionally characterized *Chlamydomonas* opsin (Cop6). It is a big protein with a cytosolic N-terminus, an 8TM opsin domain, and a large enzymatic C-terminus, containing a His kinase (DHp and CA domain), a response regulator, and a guanylyl cyclase domain. We previously could show that *Cr*2c-Cyclop1 shows guanylyl cyclase activity which is strongly inhibited by green light [8]. Another opsin, Cop5, has the same conserved domain architecture as *Cr*2c-Cyclop1. But no cyclase activity can be observed [8], probably due to a lack of key residues in the cyclase catalytic domain. However, the opsin domain of Cop5 was heterologously expressed and extensively investigated [17–20]. It was concluded that the Cop5 rhodopsin has two stable conformations with a very long lifetime that can be switched by UV-A (380 nm) and cyan (505 nm) light.

As Cop5 mRNA is present in *C. reinhardtii* [4, 5], it may be speculated that it fulfills a function in the alga, perhaps as a heterodimer with a yet unknown protein. In this study, we therefore investigated if the opsin domain of Cop5 is not only changing its absorption maximum upon illumination, but is also able to transmit a light-induced signal with a measurable output. To this end, we first constructed different chimeras of Cop5 and *Cr*2c-Cyclop1. The chimera with the opsin domain and the His kinase domain of Cop5 and the residual C-terminus of *Cr*2c-Cyclop1 (Chimera 3, see Fig. 1 and Figure S1) showed only marginal guanylyl cyclase (GC) activity (Fig. 2) which was not affected significantly by blue or UV-A illumination. This might indicate that the His kinase of Cop5 is not functional or that the phosphoryl group cannot be transferred to the response regulator domain of *Cr*2c-Cyclop1, a known prerequisite of GC activation in *Cr*2c-Cyclop1. However, we gained a light-sensitive cyclase activity by fusion of the opsin domain of Cop5 with the enzymatic domains (His kinase, response regulator, guanylyl cyclase) of *Cr*2c-Cyclop1 (Chimeras 1 and 2, see Fig. 1 and Fig. 2).

The obtained chimeras showed light-regulated guanylyl cyclase activity with two stable states of different activity. As chimera 1 showed the highest ratio of UV-A-activated to blue-inhibited activity, we investigated this chimera further and named it switch-Cyclop1. switch-Cyclop1 can be activated by 380 nm light and inhibited by blue/green light, in good agreement to the previous spectroscopic study of its rhodopsin part [17–20], and for the first time showing a functional output of this spectroscopic change. The active state after 380 nm illumination is stable for hours. switch-Cyclop1 is very sensitive to light, either to be activated by a small amount of 380 nm photons or to be 20-fold inhibited (Fig. 4d) by blue photons. We obtained a K_0.5_ of ~ 150 photons /nm^2^ of 380 nm (~ 73 J/m^2^) for activation. Less blue light photons are needed for inhibition than UV-A light photons are needed for activation. We conclude a K_0.5_ of ~ 40 photons /nm^2^ of 473 nm (~ 18 J/m^2^) for inhibition. This suggests that the blue absorbing intermediate, or the “active state,” absorbs light more efficiently.

## Conclusions

Although abundant in algal genomes, the physiological functions of 2c-Cyclop proteins are still unknown. Our study with switch-Cyclop proteins shows that the Cop5 rhodopsin can sense the UV-A/cyan ratio, which is an environmental factor influenced by daytime, weather, and water depths. However, the real light-sensing mechanism in the alga is still completely unknown. Our synthetic construct—switch-Cyclop1—is the first photosensor with enzymatic activity that can be switched on by a small amount of UV-A/violet light (380 nm photons). As it may also be switched off (to a 20-fold decreased GC activity) by application of blue or green photons, it should become a useful tool in studies on the physiological effects of cGMP. Expressing switch-Cyclop1 in the cells or tissue of interest will allow non-invasive and reversible manipulation of cGMP levels to a degree which is not possible by pharmacological intervention.

## Methods

### Generation of chimeras

Based on the pGEMHE-*Cr2c-Cyclop1* construct from our previous experiments [8], three chimeras were generated by exchanging DNA fragments between Cop5 and *Cr*2c-Cyclop1 with certain restriction sites. The vector pGEMHE-*Cr2c-Cyclop1* was digested with 5′-*Bam*HI and 3′-*Nco*I. For chimeras 1 and 2, the Cop5 part were amplified with 5′-*Bam*HI and 3′-*Ppu*MI restriction sites by PCR, and the amplified fragments were digested by the two enzymes correspondingly. Meanwhile, the other missing DNA fragment from *Cr*2c-Cyclop1 part was PCR-amplified and digested by 5′-*Ppu*MI and 3′-*Nco*I. Finally, two fragments from Cop5 and *Cr*2c-Cyclop1 were inserted to the *Bam*HI/*Nco*I cut vector in one reaction. To clone chimera 3, the DNA fragment was amplified from Cop5, digested with *Bam*HI/*Nco*I and directly ligated to the same vector.

All cloned chimeras were confirmed by complete DNA sequencing. To generate linearized DNA, plasmids were digested by NheI. cRNA was then generated after in vitro transcription by the AmpliCap-MaxT7 High Yield Message Maker Kit (Epicentre Biotechnologies).

### Expression in *Xenopus* oocyte

Thirty or 40 ng (indicated in each figure) of cRNA was injected into *Xenopus* oocytes. Injected oocytes were then incubated in ND96 buffer (96 mM NaCl, 5 mM KCl, 1 mM CaCl_2_,1 mM MgCl_2_, 5 mM HEPES, pH 7.6) supplemented with 10 μM all-trans-retinal (ATR) for 3 days at 18 °C.

### Membrane extraction and in vitro GC activity assay

After 3 days of heterologous expression in *Xenopus* oocytes, membrane extraction and in vitro reaction procedures were performed according to [8, 12, 16]. In some cases, the reactions were started by mixing 4 μl suspended membrane extract with 36 μl guanylyl cyclase reaction buffer (100 mM NaCl, 75 mM Tris-Cl pH 7.3, 5 mM MgCl_2_ (or as indicated), 5 mM DTT, 0.2 mM GTP, and 0.25 mM ATP). The reactions were performed at 20 °C or otherwise indicated in the figure. Aliquots of 10 μl reaction mix were then immediately terminated by adding 190 μl sample diluent containing 0.1 M HCl from the cGMP assay kit. After proper dilution, the cGMP concentration was determined using the DetectX High Sensitivity Direct Cyclic GMP Chemiluminescent Immunoassay Kit (Arbor assays).

### Illumination condition

Illuminations were mainly operated with lasers at 473 nm, 532 nm, 593 nm, and 635 nm wavelengths (Changchun New Industries Optoelectronics Tech) and LEDs of 380 nm (± 10 nm) and 505 nm (± 10 nm) (ProLight Opto Technology) as indicated. All experiments with blue light and UV-A light stimulation were applied with 473 nm laser and 380 nm (±10 nm) LED, respectively. Light intensities were measured with a Plus 2 optical power & energy meter (LaserPoint s.r.l, Italy).

### Data analysis

OriginPro 2017 (OriginLab Corporation, USA) and Microsoft Excel were used for data analysis. All data are shown as mean ± standard deviation (SD), as indicated respectively. Curves were fitted by the Hill1 equation in OriginPro 2017. Activation by UV-A light of dark state, Hill1 equation for Fig. 4a and b: *y* = vI + (vA − vI) × *x*^*n*/(*K*_0.5_^*n* + *x*^*n*); inhibition by blue light of fully activated enzyme, Hill1 equation for Fig. 4c and d: *y* = vA + (vI − vA) × *x*^*n*/(*K*_0.5_^*n* + *x*^*n*). Parameter definition: *y*, *v* = normalized cGMP production, vA = *v* of activated enzyme, vI = *v* of inhibited enzyme, *x* = UV-A or blue light illumination intensity (μW/mm^2^) or time (min), *K*_0.5_ = Michaelis constant, *n* = cooperative sites (Hill coefficient).

### Bioinformatics

Sequence alignment and file formatting were displayed by Clustal Omega 1.2.2 and Genedoc. The number of transmembrane helices in rhodopsin domains was predicted by integration of using web service TMHMM [21, 22] and JPred4 [23].

## Supplementary Information

**Additional file 1: Figure S1.** Sequence alignment of Cop5 and Cr2c-Cyclop1. **Figure S2.** Stability of UV-A-activated switch-Cyclop1. **Figure S3.** Activities of switch-Cyclop1 under different reaction conditions.

**Additional file 2.** Raw data values.

## Data Availability

The raw data in this study are included in the supplementary file. All the materials in this study are available from the corresponding authors on reasonable request.
