# Supplementary material for: An engineered membrane-bound guanylyl cyclase with light-switchable activity
Source: BMC Biol. 2021 Mar 29;19:54. doi: 10.1186/s12915-021-00978-6 (PMC8006352; doi:10.1186/s12915-021-00978-6)
Supplement: Supplementary file 1 — Additional file 1: Figure S1. Sequence alignment of Cop5 and Cr2c-Cyclop1. Figure S2. Stability of UV-A-activated switch-Cyclop1. Figure S3. Activities of switch-Cyclop1 under different reaction conditions. [file 12915_2021_978_MOESM1_ESM.docx]

**Supplemental figures**

**
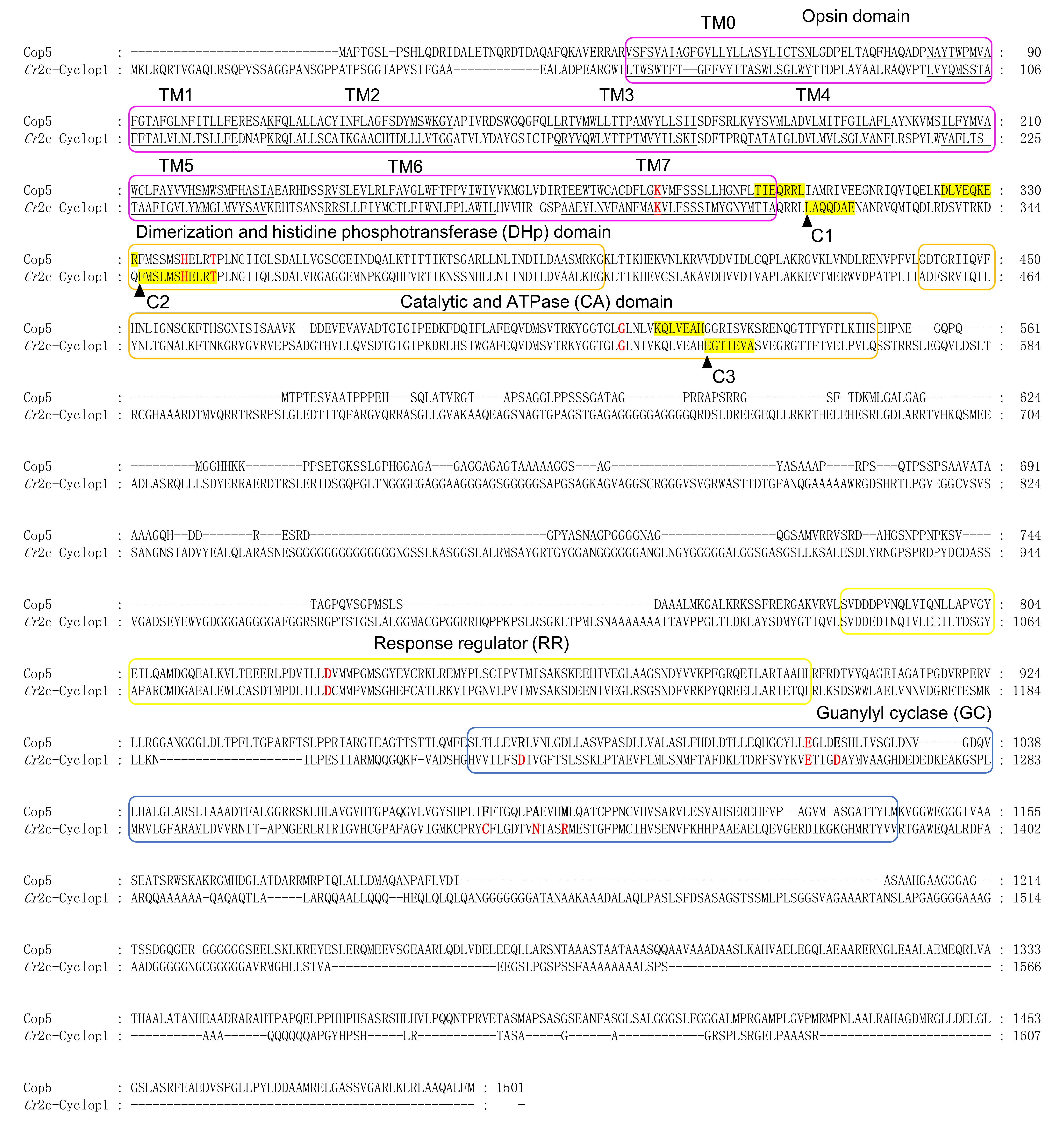
**

**Additional file 1: Fig. S1 Sequence alignment of Cop5 and *Cr*2c-Cyclop1.**

The Cop5 (Cre02.g074150) and *Cr*2c-Cyclop1 (Cre11.g467678) are aligned using Clustal Omega 1.2.2. Four conserved domains are denoted in four colored boxes, comprising of: opsin domain (purple box) with predicted 8 transmembrane helices underlined; histidine kinase (orange box, including DHp and CA domains); response regulator (RR, yellow box) and guanylyl cyclase (GC, blue box). Key residues are shown in red letters. Three black triangles are labeled as fusion points between Cop5 and *Cr*2c-Cyclop1, generating Chimera 1-3 (labeled as C1, C2 and C3).


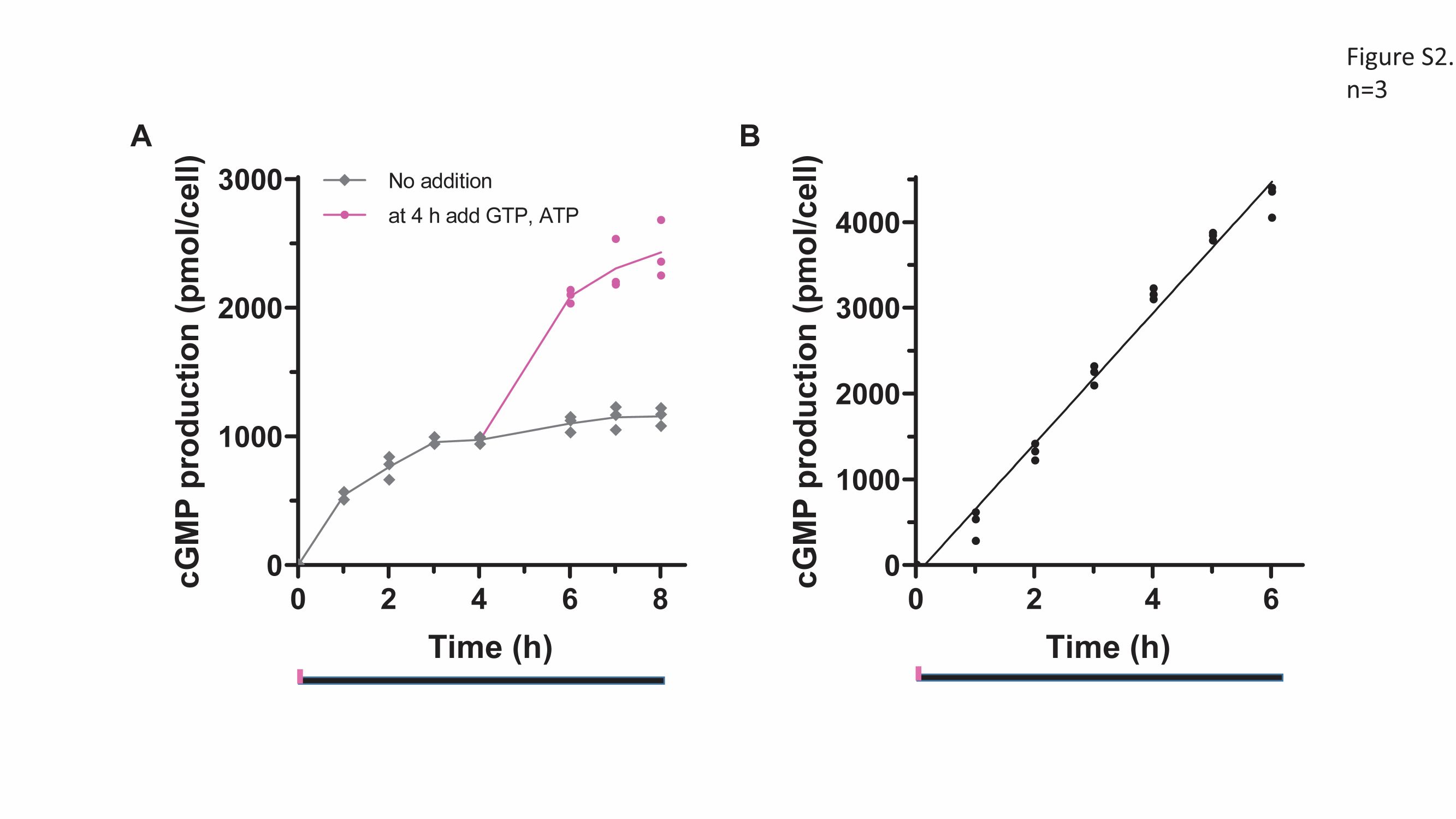


**Additional file 1: Fig. S2 Stability of UV-A-activated switch-Cyclop1.**

(A), switch-Cyclop1 activity decays with time after 30 s UV-A (380 nm, 9.6 μW/mm^2^) activation under different buffer conditions. Supplement of fresh 0.2 mM GTP, 0.25 mM ATP at 4 h restores the evoked activity.

(B), Enzyme activity is stable for 6 hours in dark after 30 s UV-A stimulation when fresh 0.2 mM GTP and 0.25 mM ATP were supplemented every hour.

For A and B, 30 ng cRNA were injected, measurements were done 3 dpi. Under graphs, the purple stroke indicated a 30 s UV-A pulse and the black line indicated the dark conditions. Results were referring to the total activities of membrane proteins extracted from one oocyte. n = 3, all data points were shown.


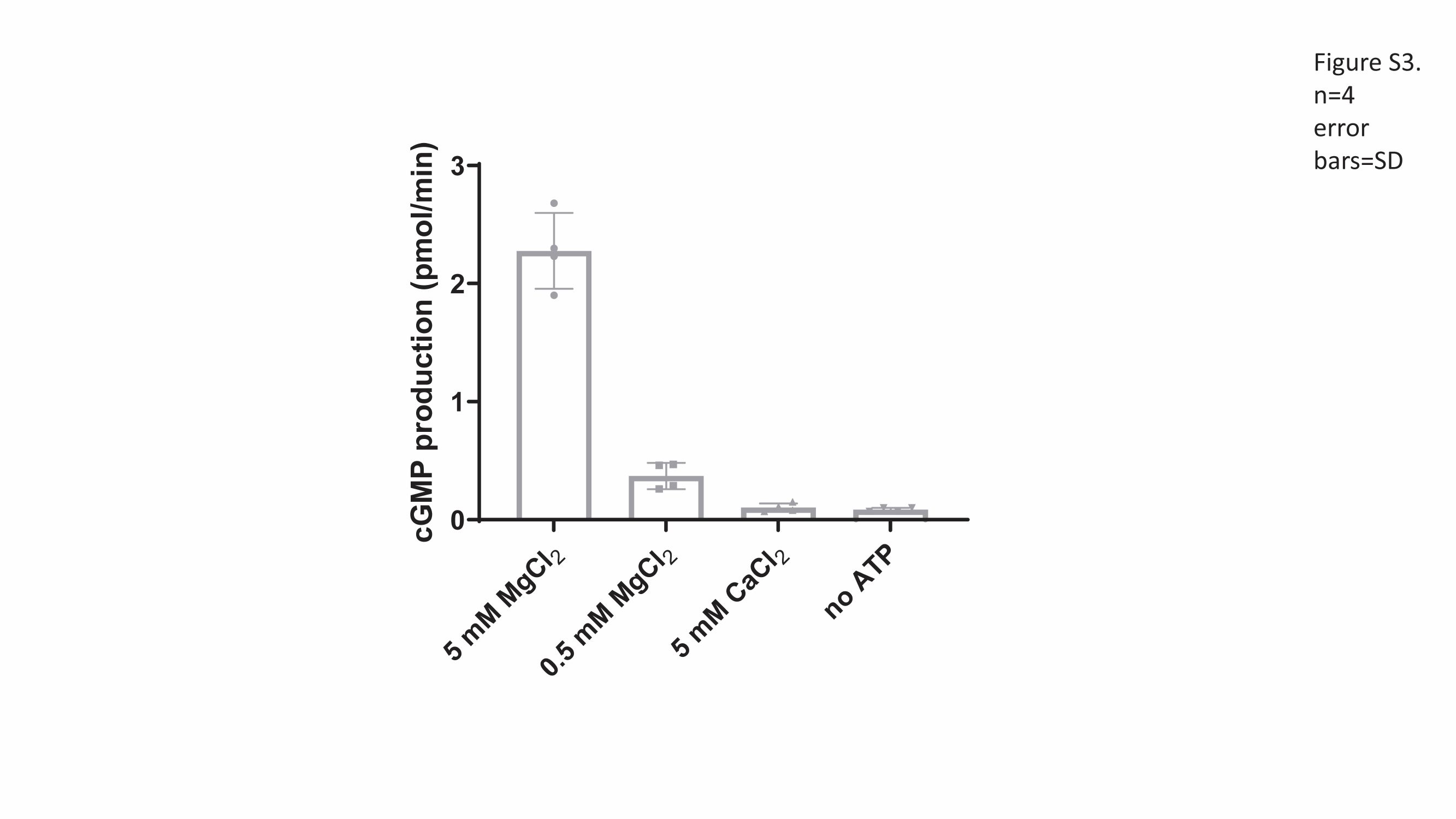


**Additional file 1: Fig. S3 Activities of switch-Cyclop1 under different reaction conditions.**

Enzyme activities detected with 5 mM MgCl_2_ (standard buffer), 0.5 mM MgCl_2_, 5 mM CaCl_2_ (replacing MgCl_2_) and without ATP. Not mentioned contents of the above buffers are the same as the standard buffer. Activities are measured for 18 min reaction in dark after 30 s UV-A (380 nm, 9.6 μW/mm^2^) illumination. 30 ng cRNA were injected, measurements were done 3 dpi (days post injection). Results were referring to the total activities of membrane proteins extracted from one oocyte. n = 4, error bars = SD.
